# Optomechanically induced transparency in multi-cavity optomechanical system with and without one two-level atom

**DOI:** 10.1038/srep28830

**Published:** 2016-06-28

**Authors:** Amjad Sohail, Yang Zhang, Jun Zhang, Chang-shui Yu

**Affiliations:** 1 School of Physics and Optoelectronic Technology, Dalian University of Technology, Dalian 116024, P.R. China

## Abstract

We analytically study the optomechanically induced transparency (OMIT) in the *N*-cavity system with the *N*th cavity driven by pump, probing laser fields and the *1*st cavity coupled to mechanical oscillator. We also consider that one atom could be trapped in the *i*th cavity. Instead of only illustrating the OMIT in such a system, we are interested in how the number of OMIT windows is influenced by the cavities and the atom and what roles the atom could play in different cavities. In the resolved sideband regime, we find that, the number of cavities precisely determines the maximal number of OMIT windows. It is interesting that, when the two-level atom is trapped in the even-labeled cavity, the central absorptive peak (odd *N*) or dip (even *N*) is split and forms an extra OMIT window, but if the atom is trapped in the odd-labeled cavity, the central absorptive peak (odd *N*) or dip (even *N*) is only broadened and thus changes the width of the OMIT windows rather than induces an extra window.

Cavity optomechanical system (OMS) has recently attracted increasing interest in both theory and experiment (ref. 1 and references therein). It usually composed of two mirrors with one fixed and the other movable or a micro-mechanical membrane oscillating inside two fixed mirrors. Such a system demonstrates the interaction between the movable oscillator and the optical field in the cavity via the radiation pressure and becomes a platform for the study^2^,^3^,^4^,^5^,^6^,^7^,^8^,^9^,^10^,^11^,^12^,^13^,^14^,^15^,^16^ of quantum ground-state cooling^17^,^18^,^19^,^20^, strong coupling dynamics^1^,^7^,^21^,^22^ and other coherent dynamics in microscopic and macroscopic domains^23^,^24^,^25^. When a strong laser field drives the optomechanical cavity, an analogue of electromagnetically induced transparency (EIT) for the output at the frequency of the weak detecting field could appear^26^,^27^. Such an EIT-like phenomenon is usually called as the optomechanically induced transparency (OMIT) which is equivalent to the case of two coupled harmonic oscillators^21^ and has been demonstrated in experiments^28^,^29^,^30^. OMIT has also been widely investigated in diverse aspects including the cases with higher-order sidebands^31^ or in the nonlinear regime^32^,^33^,^34^, OMIT in the cavity with membranes^35^,^36^ and so on. In particular, OMIT has shown many potential applications in control of light speed^29^, charge measurement^37^, single photon router^38^ and so on, which forms the further motivations to study OMIT.

Introducing the atomic freedom into OMS can not only strengthen the coupling but also allow rich physics via enhanced nonlinearities^39^,^40^,^41^. It has been applied to improve optomechanical cooling^42^,^43^,^44^ and even the ground-state cooling outside the resolved sideband regime^45^. In particular, it is shown^46^ that a two-level atomic ensemble coupled to OMS can both enhance the photon-phonon coupling through radiation pressure and broaden the transparency windows. In addition, coupled-cavity array related to the 1D waveguide or atoms has been widely studied in the control of photon transport such as quantum router^47^,^48^,^49^,^50^. Does the multiple-cavity quantum optomechanics bring new insight into OMIT? How can the OMIT be controlled if introducing the atomic freedom into the multiple-cavity system?

In this paper, we address the above questions by investigating the OMIT phenomenon in multiple-cavity optomechanical system coupled to one two-level atom. *Here instead of only illustrating the OMIT in such a system, we are especially interested in how the number of OMIT windows is related to the number of the cavities as well as the potential trapped atom and what roles the atom could play in different cavities.* Through our analytic calculations, it is shown that the maximal number of OMIT windows is precisely determined by the cavity number, if there does not exist any atom in the multi-cavity system. In particular, we find that the atom trapped in different cavities will play different roles in OMIT. When one atom is trapped in even-labeled cavity, the central absorptive peak (odd *N*) or dip (even *N*) is split and forms an extra OMIT window, but when the atom is trapped in odd-labeled cavity, the central absorptive peak (odd *N*) or dip (even *N*) is only broadened and thus changes the width of the OMIT windows instead of inducing the extra window. In addition, we also find that the multiple OMIT windows are the result of the coupling of multiple cavities irrespective of the participation of the mechanical oscillator. A numerical simulation is also given to support our results.

## Results

### The model

**Figure 1:**
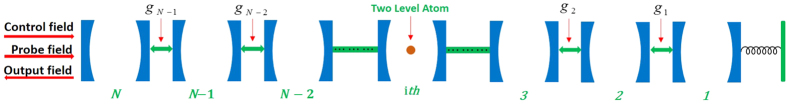
Schematic diagram of *N* cavities connected through tunneling parameters *g*_*n*_. A strong driving field and a weak probing field are injected into cavity *N* while the first cavity is coupled with mechanical resonator.

The optomechanical system under consideration is shown schematically in Fig. 1. The system includes *N* cavities labelled by 1, 2, ···, *N* with the frequency of *j*th cavity denoted by *ω*_*j*_. The *n*th and (*n* + 1)th cavities with *n* ≠ *N* are connected through tunneling parameters (hopping rates) *g*_*n*_. Such a coupled cavity array (2D) has been systematically studied in various cases in ref. 51 and later considered in the single-photon router^49^,^50^. Here we only consider 1D cavity chain, in particular, we let one end mirror of Cavity 1 be movable as shown in Fig. 1. Thus it forms an optomechanical system. Cavity *N* is separately driven by one coupling field *ε*_*c*_ and one probing field *ε*_*p*_. In addition, we assume that one two-level atom could be trapped in the *i*th cavity 1 ≤ *i* ≤ *N* with *g*_*a*_ denoting the atom-cavity coupling strength. In this model, the optical modes are described by annihilation (creation) operators 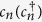 and the mechanical mode is represented by *b*(*b*^†^) which is equivalent to the description by *x*_*m*_ and *p*_*m*_. This similar description can be found in ref. 41. Let the frequency of the coupling field be *ω*_*c*_, so in the rotating frame at *ω*_*c*_, the Hamiltonian of our system reads


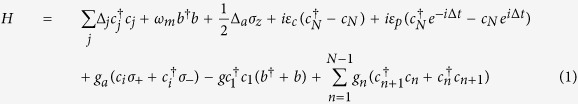


with *ω*_*p*_, *ω*_*a*_ representing the frequency of the probing field and the atomic transition frequency. In Eq. (1) the first three terms, respectively, denote the free Hamiltonian for the cavities, the movable mirror and the trapped atom with Δ_*j*_ = *ω*_*j*_ − *ω*_*c*_ Δ = *ω*_*p*_ − *ω*_*c*_ and Δ_*a*_ = *ω*_*a*_ − *ω*_*c*_, the last two terms in first line corresponds to the interaction of the *N*th cavity driven by the coupling field *ε*_*c*_ and the probing field *ε*_*p*_. The first term in the second line of Eq. (1) describes the interaction between the atom and the *i*th cavity, the second term corresponds to the interaction between the *1*st cavity and the movable mirror via the radiation pressure, and the last term describes the hopping between the two adjacent cavities. In addition, *g* in Eq. (1) denotes the coupling strength between the 1*st* cavity and the mechanical oscillator. It is obvious that Δ_*a*_ = *g*_*a*_ = 0 means no atom in the cavities.

### The dynamics

Based on the above Hamiltonian, one can easily obtain the Langevin Equations for the operators. So the corresponding equations for the mean value of operators in the mean-field approximation, viz, 〈*st*〉 = 〈*s*〉〈*t*〉, can be given by


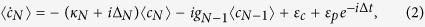



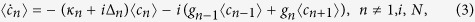



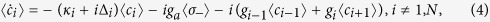



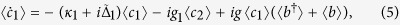



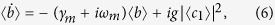



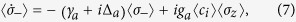



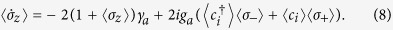


Here *κ*_*n*_ denotes the leakage of *n*th cavity and *γ*_*m*_ and *γ*_*a*_, respectively, represent the decay rates of the mechanical oscillator and the atom. If the atom is trapped in the first cavity, Eq. (5) should be replaced by


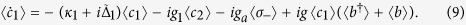


If the atom is trapped in *N*th cavity, Eq. (2) should be replaced by


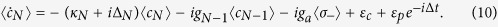


In order to solve the dynamics, we suppose


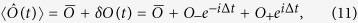


for any operator 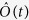 with 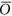 denoting the steady-state value without *ε*_*p*_ and *δO* = *O*_−_*e*^−*i*Δ*t*^ + *O*_ + _*e*^*i*Δ*t*^ induced by the weak probing field. Substituting Eq. (11) into Eqs (2, 3, 4, 5, 6, 7, 8, 9, 10), one can obtain an equation array for 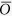 which has the same form as Eqs (2, 3, 4, 5, 6, 7, 8, 9, 10) except *ε*_*p*_ = 0 and 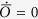. This equation arrays are omitted here. In addition, one can also obtain an equation array for *δO*(*t*) which is given in the Methods (Eqs (17, 18, 19, 20, 21, 22)). By solving the equations for 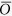, one can find that


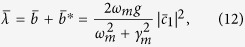


and


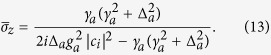


In addition, considering Eq. (11), one can easily find the equations for *O*_±_. However, for the purpose of this paper, we only provide the equations for *O*_−_ in the Methods Eqs (23, 24, 25, 26, 27, 28) within the resolved sideband regime, i.e., *ω*_*m*_ ≫ *κ* and 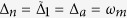 where 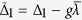. These equations provide the fundamental description of the dynamics of the model considered here.

### Output field

In order to reveal the OMIT, we will have to find out the response of the system to the probing frequency, which can be detected by the output field. Based on the input–output theory^52^, we can obtain


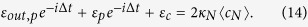


Substituting Eq. (11) into Eq. (14), one can find that the total output field at the probing frequency *ω*_*p*_ can be given by


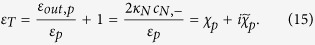


It is clear that *χ*_*p*_ = Re(*ε*_*T*_) and 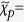 Im(*ε*_*T*_) are the in-phase and out-of-phase quadratures of the output probing field, representing the absorptive and dispersive behavior of the output probing field, respectively. The quadrature can be measured via the homodyne technique^52^. So the next task is to find *c*_*N*,−_. In order to gain more physical insight, we only consider the system in the sideband resolved regime. Thus *c*_*N*,−_ can be easily obtained by solving Eqs (23, 24, 25, 26, 27, 28). So the output field *ε*_*T*_ can be directly given by


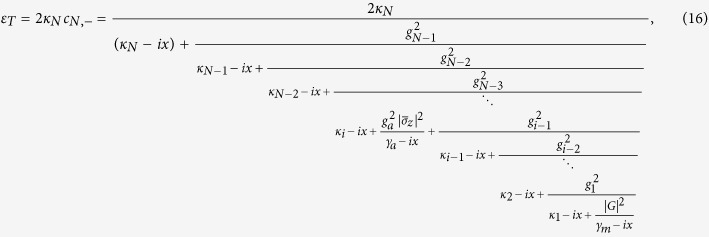


where *x* = Δ − *ω*_*m*_ and 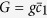 is the effective optomechanical rate. In above equation, the first line of the denominator represents two cavities with radiative decays *κ*_*N*_ and *κ*_*N*−1_ are connected through their coupling strength *g*_*N*−1_. Second line represents two cavities with radiative decays *κ*_*N*−1_ and *κ*_*N*−2_ are connected through their coupling strength *g*_*N*−2_ and so on. The *1*st cavity in the last line is coupled to the mechanical oscillator by an effective coupling *G*. In addition, an extra term 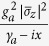 corresponding to the atomic contribution appears in the *κ*_*i*_ line which denotes the atom is coupled to the *i*th cavity with an effective coupling 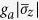. Certainly, if the atom is trapped in the first cavity, this term will appear in the last line. If the atom is placed in the *N*th cavity, it will appear in the first line of the denominator. It is obvious that the output field depends on both the parameters of the system and the steady-state values of *c*_1_ and *c*_*i*_. These two values can be determined by solving the equations for all 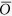 which have been omitted here. But the concrete expressions of *c*_1_ and *c*_*i*_ are quite complicated, so it is impossible to present the concrete forms. It is fortunate that this does not influence our understanding on the OMIT window numbers. One can find from the latter part that the values of *c*_1_ and *c*_*i*_ only affect the width of the OMIT windows. In this sense, it doesn’t matter whether they can be simultaneously assigned by some values. Therefore, for simplicity, one can select that 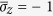 and *G* can be given by any reasonable and convenient assignment.

### OMIT windows

The OMIT is signaled by the simultaneously vanishing absorption and dispersion, which is further related to the simultaneously vanishing *χ*_*p*_ and 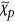, that is *ε*_*T*_. In order to show the OMIT windows as many as possible, we restrict ourselves to the weak dissipative regime, i.e., 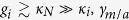, to discuss the points where *ε*_*T*_ vanishes. This is also supported by our latter numerical procedures.

#### Without atom

**Figure 2:**
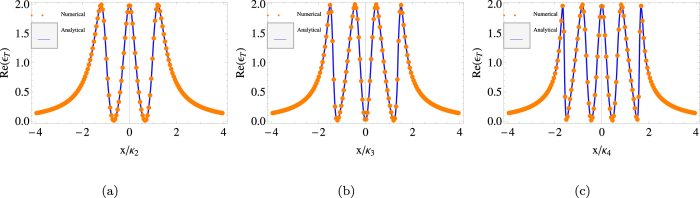
The Real part Re(*ε*_*T*_) in the absence of atom-field coupling, as a function of *x*/*κ*_*N*_ for (**a**) two cavities, (**b**) three cavities and (**c**) four cavities with the parameters *ω*_*m*_ = 2*π* × 51.8 *MHz*, *γ*_*m*_ = 2*π* × 41 *KHz*, *G* = 2*π* × 10 *Mz*, *κ*_*N*_ = 2*π* × 15 *MHz*, *κ*_1_ = *κ*_2_ = *κ*_3_ = ··· = 2*π* × 0.027 *MHz*, and the coupling rates *g*_1_ = *g*_2_ = *g*_3_ = *κ*_*N*_. The solid lines show the analytic expressions given by Eq. (16), but the dotted lines represent the solution numerically solved from Eqs (23, 24, 25, 26, 27, 28), which guarantees the validity of our analytical result Eq. (16).

**Figure 3:**
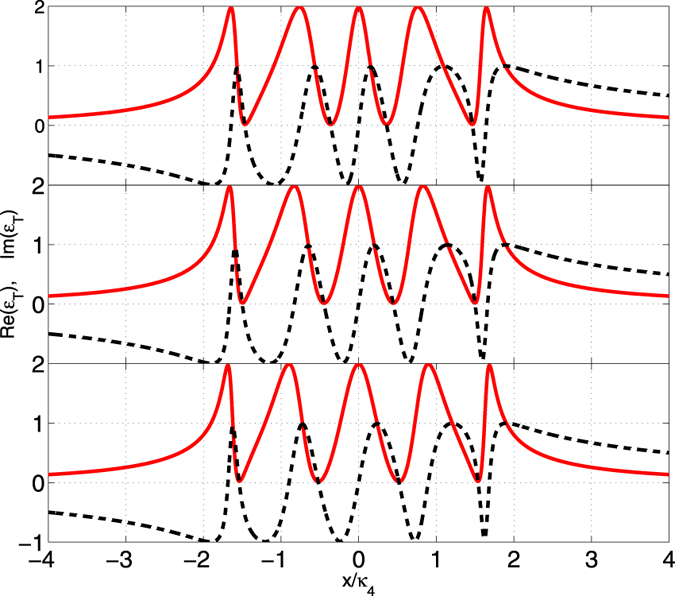
Real part Re(*ε*_*T*_) (solid red) and the imaginary part Im(*ε*_*T*_) (dashed black) as a function of *x*/*κ*_4_ for four cavities. The three subplots from above to bottom, respectively, correspond to *G* = 8 *MHz*, *G* = 10 *MHz* and *G* = 12 *MHz*. The other parameters are the same as in Fig. 2. One can see that the width of the central absorptive peak becomes wide with the increasing of *G*.

If there does not exist any atom in the optomechanical system, the term with *g*_*a*_ vanishes due to *g*_*a*_ = 0. In this case, the vanishing *ε*_*T*_ means that the denominator approaches infinity which can be further determined by the vanishing denominator corresponding to the numerator |*g*_*N*−1_|^2^. It is obvious that the condition with such a vanishing denominator corresponds to an equation with *N* degrees. Therefore it has at most *N* different roots. This means that such an optomechanical system has at most *N* OMIT windows. To give an intuitive illustration of the OMIT, we numerically evaluate OMIT and demonstrate the multiple transparency windows due to the interaction between cavity fields and the mechanical oscillator. We take the parameters from^53^,^54^,^55^ where, the damping rate of mechanical oscillator *γ*_*m*_ = 2*π* × 41 kHz, decay rate of the driven cavity field *κ*_*N*_ = 2*π* × 15 MHz and the frequency of oscillator *ω*_*m*_ = 2*π* × 51 MHz. For the case of the resolved sideband regime, i.e. the mechanical frequency is much greater than the decays and 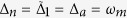, we plot the phase quadratures of the output probing fields for a system with two, three and four cavities in Fig. 2 which exhibits two windows, three windows and four windows respectively. We assume that the 1*st* cavity coupled to mechanical oscillator with *G* = 2*π* × 12 MHz. The multiple transparency windows display that the optomechanical system becomes simultaneously transparent to the probing field at multiple different frequencies, which is the result of the destructive interferences between the input probing field and the anti-Stokes fields generated by the interactions of the coupling field with the multiple cavities. In addition, in order to show the effects of *G*, we plot Fig. 3 with different choices of *G*. One can find that the larger *G* corresponds to the wider central absorptive peak (or dip for odd number of cavities) in the valid range of *G*. Numerical results show that the interval that the OMITs occur (from about −2 to 2 in all the figures) is almost independent of the numbers of cavities. In fact, the width is determined by all the hopping rate *g*_*n*_. Here in order to find out many enough OMIT windows, we let all *g*_*n*_ = *κ*_*N*_, so the interval (if defined by the half width) is slightly changed. Under this condition, by numerical demonstrations, we find that the half width is increased with *N*. In particular, one can easily prove that when *N* tends to infinity, the half width is just 4. So when the central absorptive peak or dip gets wider, and the others get narrower due to the fixed interval. In one word, the value of *G* only affects the width of the transparency window instead of the maximal number of the OMIT window.

#### One atom in one cavity

Since we have set 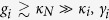, for an intuitive understanding of the number of OMIT windows, one can safely neglect the dissipative constants which contributes to the level width of the cavity as well as the atom. Under such a condition, one can find that there exist two cases in our optomechanical system.

**Figure 4:**
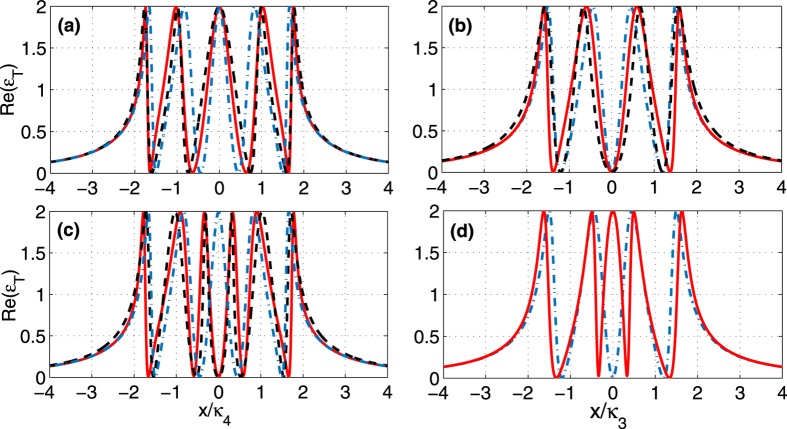
Real part Re(*ε*_*T*_) of the output field as a function of *x*/*κ*_3/4_ with γ_*a*_ = 2*π* × 0.01 *MHz* and g_*a*_ = 2*π* × 10 *MHz*. The other parameters are the same in Fig. 2. (**a,c**) illustrate cases with four cavities, where (**a**) corresponds to the atom trapped in cavity 1 (solid red) and cavity 3 (dashed black) and (**c**) corresponds to the atom in cavity 2 (solid red) and cavity 4 (dashed black). (**b,d**) correspond to the cases with three cavities, where (**b**) illustates the atom trapped in cavity 1 (solid red) and cavity 3 (dashed black) and (**d**) shows the atom trapped in cavity 2 (solid red). The dashed blue lines in all the figures mean no trapped atom.

 1) **The atom is trapped in the odd-labeled cavity.** In this case, one can see that the extra term 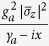 can only exist in the lines corresponding to *κ*_1_, *κ*_3_, ···. The contribution of such an extra atomic term is mathematically to increase the numerator of the same line and physically to directly broaden the central absorptive peak for even *N* (or absorptive dip for odd *N*) and then to change the width of the OMIT windows, which is analogous to increasing *G* in the case without atom. The most obvious example is when the atom is trapped in the first cavity. One can easily find that for weak *γ*_*a*_ and *γ*_*m*_, the atomic term can be approximately absorbed in the term corresponding to the mechanical oscillator and the net result is equivalent to increasing |*g*_1_|^2^. 2) **The atom is trapped in the even-labeled cavity.** In this case, the extra atomic term can lead to that the degree of the equation of the vanishing denominator corresponding to the numerator |*g*_*N*−1_|^2^ is added by 1. So when the atom is trapped in the even-labeled cavity, one can find one more extra OMIT window compared with the case without any atom. Similarly, in order to give an illustration of these different cases, we numerically evaluate the OMIT. We plot the figure in Fig. 4 with *g*_*a*_ = 2*π* × 10 MHz and *γ*_*a*_ = 2*π* × 0.01 MHz. However, we don’t plot the imaginary part Im(*ε*_*T*_) for the sharp illustration. We observe that, in four-cavity system, the width of the central absorptive peak tends to become wide through embedding the atom into the cavity 1 or cavity 3 as shown in Fig. 4(a). But, when the atom is placed in cavity 2 or 4, we have found the resonant character of the weak probing field changes and the central absorptive peak splits. Hence four OMIT windows transfigure to a penta OMIT window, as shown in Fig. 4(c). Similarly, in Fig. 4(b,d) that correspond to the cases of three cavities, one can find that the atom will directly lead to the broadening or splitting of the central absorptive dips instead of absorptive peaks.

#### The role of the mechanical oscillator

Actually the physical mechanism of the mechanical oscillator about the production of OMIT has been well known^26^,^27^. In this part, we are only interested in how the existence of the mechanical oscillator affects the number of OMIT windows. If there does not exist any mechanical oscillator, that means *G* = 0. If the atom is trapped in the first cavity under this condition, the number of the OMIT windows will keep invariant, but the width of the OMIT window will become narrow. This could be equivalently understood as the case without atom in the optomechanical system. That is, the role of the mechanical oscillator is to broaden the OMIT window in this case. In other cases, that is, no atom exists or the atom is only trapped in the even-labeled cavity and so on, one can easily find that the OMIT windows will be decreased by 1. In this case, one can draw the conclusion that the mechanical oscillator contributes an OMIT window. In this sense, we can say that the multiple OMIT windows should come from the coupling of the multiple cavities instead of the direct participation of the mechanical oscillator.

## Discussions and Conclusion

Before the end, we would like to emphasize that similar to multiple EIT windows, the multiple OMIT windows permit the probing light with different frequencies to transmit simultaneously. So the OMIT with multiple windows could also be used in multi-channel optical communication and multichannel quantum information processing^56^. OMIT is also closely related to the superluminal and ultraslow light propagation^18^,^57^, the quantum router^38^, charge measurement^37^ and so on. Hence, OMIT with multiple transparency windows could mean wider applications. In addition, the experimental realization of coupled cavity array is systematically reviewed in ref. 51. The parameters we used are mainly taken from refs 18, 28 and 29 which report the current experiments about the optomechanical system and OMIT. These can be used to well evaluate the feasibility.

We would also like to mention that one can also consider an atomic ensemble instead of a single atom in the system. We think that the net effect is equivalent to increasing the coupling between the single atom and cavity if the atomic ensemble is considered in the limit of large atomic number. In addition, if two or more atoms are trapped in different even-labeled cavities, respectively, we think multiple extra windows will occur. If they are trapped in different odd-labeled cavities, the OMIT window will change much greater. In addition, the entanglement in this optomechanical system is an interesting topic. Our preliminary results have shown the entanglement can be produced between the different components of this optomechanical system (such as between two cavities, or between one cavity and the movable mirror). It is interesting that the entanglement between the mirror and the *N*th cavity could be enhanced by the multiple cavities, but the entanglement of other components could also be reduced. In particular, the existence of the atom could play different roles in the control of the generation of various entanglement. All the detailed results will be reported in the latter papers.

In summary, we have theoretically discussed the response of an optomechanical system which includes *N* cavities. We have given a general analytical expression of the generation of multiple OMIT windows. The mechanism of OMIT could have been well understood and even one could have known that an atom or atomic ensemble could broaden the width of OMIT window. However, it is shown here that the number of the OMIT windows directly depend upon the number of cavities. In particular, we find that, when the atom is trapped in even-labeled cavity, the number of the OMIT windows will be increased by one; if the atom is trapped in the odd-labeled cavity, the only the width of the OMIT windows could be changed. In addition, we also find that the multiple OMIT windows are only attributed to the coupling of the multiple cavities and irrespective of the coupling to the mechanical oscillator, because the mechanical oscillator could produce only one additional OMIT window or change the width of the OMIT windows which depends on the even- or odd- labelled cavity that the atom is trapped in.

## Methods

In this section, we will give a brief introduction of the derivation of the equations used in the main text. Substituting 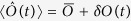 given in Eq. (11) into Eqs (2, 3, 4, 5, 6, 7, 8, 9, 10), Eqs (2, 3, 4, 5, 6, 7, 8, 9, 10) can be rewritten by 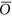 and *δO*(*t*). Since *δO*(*t*) is small and depends on time and 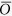 is independent of time. One can separate equations into one related to time and the other irrelevant of time. The equation array irrelevant of time corresponding to 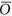 has the same form as Eqs (2, 3, 4, 5, 6, 7, 8, 9, 10) except setting *ε*_*p*_ = 0 and 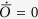. In other words, if we replace 〈*O*〉 in Eqs (2, 3, 4, 5, 6, 7, 8, 9, 10) by 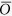 and let *ε*_*p*_ = 0 and 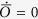, we will obtain the equations for 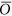. Our Eqs (12) and (13) are solved from these equations, but for avoiding repetition, these equations are omitted here. The equations with time corresponding to *δO*(*t*) should obviously include the term *ε*_*p*_*e*^−*i*Δ*t*^. They can be directly given as follows.


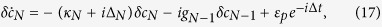



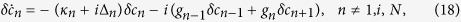



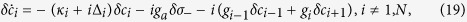



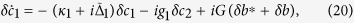



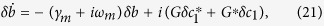



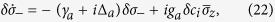


where 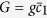 is the effective optomechanical rate. As mentioned in the text, we consider the system in the resolved sideband regime in order to gain more physical insight. That is, we let *ω*_*m*_ ≫ *κ* and 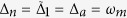. In such a resolved sideband regime, the lower sideband, far off-resonance can be safely neglected. This means that in Eq. (11), *O*_+_ ≈ 0 which is the same as^28^. Thus, Eqs (17, 18, 19, 20, 21, 22) can be rewritten for *O*_−_ as


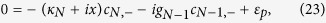



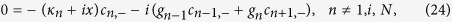



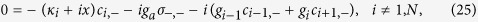



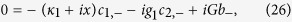



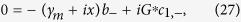



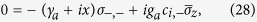


where *x* = Δ − *ω*_*m*_ is again the detuning from the center line of the sideband.

## Additional Information

**How to cite this article**: Sohail, A. *et al*. Optomechanically induced transparency in multi-cavity optomechanical system with and without one two-level atom. *Sci. Rep.* **6**, 28830; doi: 10.1038/srep28830 (2016).
